# Association between non-scarring alopecia and hypothyroidism: a bidirectional two-sample Mendelian randomization study

**DOI:** 10.3389/fendo.2024.1356832

**Published:** 2024-03-18

**Authors:** Jiankang Yang, Zhenlai Zhu, Chen Zhang, Yanyang Guo, Gang Wang, Meng Fu

**Affiliations:** ^1^ Department of Dermatology, Xijing Hospital, Fourth Military Medical University, Xi’an, China; ^2^ Xijing 986 Hospital Department, Fourth Military Medical University, Xi’an, China

**Keywords:** non-scarring alopecia, alopecia areata, androgenic alopecia, hypothyroidism, Mendelian randomization

## Abstract

**Background:**

Non-scarring alopecia is typically represented by two main types: alopecia areata (AA) and androgenetic alopecia (AGA). While previous observational studies have indicated a link between non-scarring alopecia and hypothyroidism, the precise causal relationship remains uncertain. To determine the potential links between non-scarring alopecia and hypothyroidism, we conducted a bidirectional two-sample Mendelian randomization (MR) analysis.

**Methods:**

We used independent genetic instruments from the FinnGen consortium for AA (682 cases, 361,140 controls) and AGA (195 cases, 201,019 controls) to investigate the association with hypothyroidism in the UK Biobank study (22,687 cases, 440,246 controls). The primary analysis was performed using the inverse variance-weighted method. Complementary approaches were employed to evaluate the pleiotropy and heterogeneity.

**Results:**

Genetically predicted AA exhibited a positive causal effect on hypothyroidism (odds ratio [OR], 1.0017; 95% confidence interval [CI], 1.0004-1.0029; *P* = 0.0101). Additionally, hypothyroidism was found to be strongly correlated with an increase in the risk of AA (OR, 45.6839; 95% CI, 1.8446-1131.4271, *P* = 0.0196). However, no causal relationship was demonstrated between AGA and hypothyroidism. A sensitivity analysis validated the integrity of these causal relationships.

**Conclusion:**

This MR study supports a bidirectional causal link between AA and hypothyroidism. Nevertheless, additional research is needed to gain a more thorough comprehension of the causal relationship between non-scarring alopecia and hypothyroidism.

## 1 Introduction

Alopecia is a widespread and troubling problem that can directly affect an individual’s self-esteem and quality of life. Non-scarring alopecia is the most commonly occurring type, with alopecia areata (AA) and androgenetic alopecia (AGA) accounting for the majority of cases (1). AA is an autoimmune condition characterized by patchy, sometimes relapsing hair loss (2). It impacts around 2% of the overall population at some stage during their lifetime (3). Although often referred to as male-pattern alopecia, AGA is the predominant form of scalp hair loss affecting both men and women. It is distinguished by an ongoing miniaturization of the follicular unit, typically following a distinctive pattern of dispersion (4).

Non-scarring alopecia is a complex process involving genetic predisposition, environmental triggers, and endocrine factors, including thyroid hormone, which may contribute to its pathological changes (5). Hypothyroidism, a common thyroid dysfunction, is identified by an elevated serum thyroid-stimulating hormone level and a reduced thyroxine level (6). Previous studies proposed a significant association between hypothyroidism and non-scarring alopecia, particularly AA (7–9). However, due to the heterogeneous methodology and observational design, some cross-sectional, case-control, and cohort studies as well as meta-analyses even produced conflicting results (7–13). Hence, the connection between non-scarring alopecia and hypothyroidism warrants further investigation.

Mendelian randomization (MR) is an analytic method that employs genetic variants as instrumental variables (IVs) to represent potential risk factors (14). These genetic variants are randomly assigned during conception, thus excluding reverse causation and confounding bias inherent in observational studies (14). MR analysis can provide unbiased estimates of disease risk in the absence of pleiotropy (14). Here, we used a MR analysis to explore the causal relationship between non-scarring alopecia and hypothyroidism.

## 2 Methods

### 2.1 Study design

To establish the causality between non-scarring alopecia and hypothyroidism, we performed bidirectional two-sample MR analyses utilizing summary statistics from genome-wide association studies (GWASs). Ethical approval and informed consent were not needed for this study because it used the summary-level publicly available GWAS data, and all of the original studies had already met these approval requirements. The report follows the guidelines for Strengthening the Reporting of Observational Studies in Epidemiology - Mendelian Randomization (15).

### 2.2 Data sources

GWAS summary statistics for AA and AGA (according to the international classification of diseases [ICD] codes) was selected from the FinnGen Consortium R9, including 361,822 (682 AA cases and 361,140 controls) and 201,214 (195AGA cases and 201,019 controls) European-descent participants, respectively (https://www.finngen.fi/en) (**Supplementary Table 1**). For hypothyroidism, summary statistics of a GWAS including 22,687 subjects from the UK Biobank (UKB) with hypothyroidism (according to self-reported history of hypothyroidism/myxedema) and 440,246 controls were considered (https://gwas.mrcieu.ac.uk/datasets/ukb-b-19732/) (**Supplementary Table 1**). The age at diagnosis for self-reported hypothyroidism ranged from 0 to 70 years. Demographic information about participants, including age and gender, is available in the FinnGen project (https://risteys.finngen.fi/). The FinnGen and UKB GWAS datasets have little overlap, which ensures a low type 1 error rate for both forward and reverse MR.

### 2.3 Genetic IVs selection

To ensure reliable estimation of causal effects, the single nucleotide polymorphisms (SNPs) employed as IVs in the MR analysis must meet three requirements: relevance (highly associated with the exposure), independence (no shared common cause with the outcome), and exclusion restriction (only influences the outcome via the exposure pathway) (16, 17).

In this study, SNPs were utilized as IVs at the genome-wide significance threshold (*P* =5×10^-8^). However, due to the limited number of SNPs associated with non-scarring alopecia at the threshold, a less stringent significance level (AA: *P* =1×10^-5^, AGA: *P* =5×10^-5^) was recommended. To establish the independence of SNPs, IVs were clumped within a 10 Mb genetic window at a stringent linkage disequilibrium (LD) threshold (*r^2 ^= *0.001). The next step was to harmonize the effect estimates for both exposure and outcome variants. Potential palindromic SNPs were eliminated. When the outcome datasets did not contain certain SNPs, we used an appropriate proxy in strong LD with *r^2 ^= *0.8. Subsequently, we screened the PhenoScanner database (http://www.phenoscanner.medschl.cam.ac.uk/phenoscanner) to exclude SNPs that could potentially affect the outcome. For example, all SNPs related to autoimmune diseases (e.g., lupus erythematosus, and vitiligo), allergic diseases (e.g., atopic dermatitis, and allergic rhinitis), and mental disorders (e.g., stress, anxiety, and depression) that could affect AA (18, 19) when considering the outcome of MR were eliminated. The *F*-statistics and variance (*R^2^*) were used to assess the strength of the IVs for the selected SNPs to avoid weak-tool bias (20). Furthermore, we employed the MR-Egger intercepts (21) and MR pleiotropy residual sum and outlier (MR-PRESSO) (22) method to identify and eliminate outliers.

### 2.4 MR analyses

Bidirectional two-sample MR was performed using four methods, namely MR-Egger (23), weighted median (WM) (24), inverse variance weighted (IVW) (25) and robust adjusted profile score (RAPS) (26), with IVW being the predominant approach. If heterogeneity was present, a random effects IVW model was utilized, otherwise a fixed-effect model was applied (27). Heterogeneity was examined by Cochran’s Q test with the IVW and MR-Egger methods (28). MR-Egger intercepts (21) and MR-PRESSO framework (22) were used to assess the potential directional and horizontal pleiotropy, respectively. Additionally, we conducted a leave-one-out analysis to determine if any individual outlier variant influenced the effect estimates (26).

We performed MR analyses with the statistical software R (version 4.3.1) and the TwoSampleMR (version 0.5.7) and MR-PRESSO (version 1.0) packages. The results were reported as odds ratios (ORs) with 95% confidence intervals (CIs) and were considered statistically significant at a threshold of *P* < 0.05. If the IVW method shows significance, even if the other methods are inconclusive, it may be considered a positive finding, as long as the β values from the other methods are in the same direction without pleiotropy or heterogeneity (29).

## 3 Results

### 3.1 Casual effect of non-scarring alopecia on hypothyroidism

**Figure 1:**
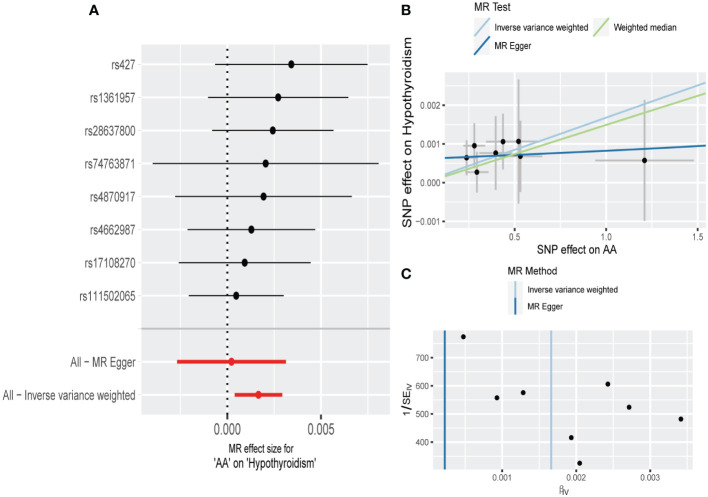
Mendelian randomization (MR) analysis of causal effect of alopecia areata (AA) on hypothyroidism. **(A)** The forest plot displays individual estimates of hypothyroidism risk for each single nucleotide polymorphism (SNP) as black dots, and pooled estimates as red dots. **(B)** The scatter plot illustrates the genetic association between the risk of AA on the x-axis and the risk of hypothyroidism on the y-axis. Each line indicates the causal relationship for each approach. **(C)** Funnel plot shows the overall heterogeneity of MR estimates.

Following strict exclusion criteria, we identified 8 SNPs for AA, 23 SNPs for AGA as IVs. The *F*-statistics for all of these IVs were greater than 10, implying the absence of weak instrument bias (**Supplementary Table 2**). According to the IVW analysis, there is weak evidence of a potential causal effect of AA on the risk of hypothyroidism, with borderline statistical significance (OR, 1.0017; 95%CI, 1.0004-1.0029; *P* = 0.0101; **Table 1**, **Figure 1**) were observed. This was confirmed with RAPS method (OR, 1.0017; 95%CI, 1.0003-1.0031; *P* = 0.0167; **Table 1**). The MR-Egger and WM estimates were consistent with IVW in the same direction, though without statistical significance (**Table 1**, **Figure 1**). However, IVW, RAPS, WM, and MR-Egger, all failed to support a causal association of AGA with hypothyroidism (**Table 1**, **Supplementary Figure 1**).

**Table 1:** Bidirectional MR estimates for the associations between non-scarring alopecia and hypothyroidism.

Exposure	Outcome	Methods	nSNPs	β	OR	95%CI	*P*
AA	Hypothyroidism	MR Egger	8	0.0002	1.0002	0.9973-1.0031	0.8843
		WM	8	0.0015	1.0015	0.9999-1.0031	0.0638
		IVW	8	**0.0017**	**1.0017**	**1.0004-1.0029**	**0.0101**
		RAPS	8	**0.0017**	**1.0017**	**1.0003-1.0031**	**0.0167**
Hypothyroidism	AA	MR Egger	95	1.4234	4.1511	0.0003-54020.7604	0.7690
		WM	95	3.5223	33.8633	0.2444-4692.6806	0.1615
		IVW	95	**3.8217**	**45.6839**	**1.8446-1131.4271**	**0.0196**
		RAPS	95	**4.0751**	**58.8579**	**2.3497-1474.344**	**0.0131**
AGA	Hypothyroidism	MR Egger	23	0.0005	1.0005	0.9993-1.0018	0.4074
		WM	23	-0.0003	0.9997	0.9990-1.0004	0.4343
		IVW	23	-0.0003	0.9997	0.9993-1.0002	0.3143
		RAPS	23	-0.0002	0.9998	0.9996-1.0001	0.1571
Hypothyroidism	AGA	MR Egger	112	0.7937	2.2115	4.84×10^-5^-1.01×10^5^	0.8850
		WM	112	-2.2777	0.1025	2.33×10^5^-450.9772	0.5946
		IVW	112	0.7087	2.0313	0.0157-262.1790	0.7750
		RAPS	112	0.4107	1.5080	0.0120-189.3998	0.8677

MR, Mendelian randomization; AA, alopecia areata; AGA, androgenetic alopecia; nSNPs, number of single nucleotide polymorphisms; OR, odds ratio; CI, confidence interval; WM, weighted median; IVW, inverse-variance weighted; RAPS, robust adjusted profile score. Bold: OR ≥1.0 and *P* < 0.05.

These results were supported by an absence of horizontal pleiotropy, indicated by the non-significant results of MR-Egger regression and MR-PRESSO (**Table 2**, **Figure 1**, **Supplementary Figure 1**). The Cochran’s Q test conducted on IVW and MR-Egger, along with the examination of funnel plots, did not reveal any evidence of heterogeneity (**Table 2**, **Figure 1**, **Supplementary Figure 1**). In addition, upon removing each SNP consecutively, no significant SNP was identified by leave-one-out analysis (**Supplementary Figures 3A, B**).

**Table 2:** Sensitivity analyses of MR after outlier correction.

Exposure	Outcome	Heterogeneity test				Pleiotropy test			
		IVW		MR-Egger		MR-Egger regression		MR-PRESSO	
		*Q*	*P*	*Q*	*P*	Intercept	*P*	RSS	*P*
AA	Hypothyroidism	2.321	0.940	1.155	0.979	6.10×10^-4^	0.322	3.383	0.939
Hypothyroidism	AA	95.957	0.424	95.670	0.404	0.010	0.599	99.957	0.415
AGA	Hypothyroidism	25.584	0.270	23.514	0.317	-5.20×10^-4^	0.188	43.468	0.125
Hypothyroidism	AGA	112.452	0.444	112.452	0.417	-4.34×10^-4^	0.986	115.767	0.432

MR, Mendelian randomization; AA, alopecia areata; AGA, androgenetic alopecia; IVW, inverse-variance weighted; MR-PRESSO, MR pleiotropy residual sum and outlier; RSS, residual sum of squares.

### 3.2 Casual effect of hypothyroidism on non-scarring alopecia

**Figure 2:**
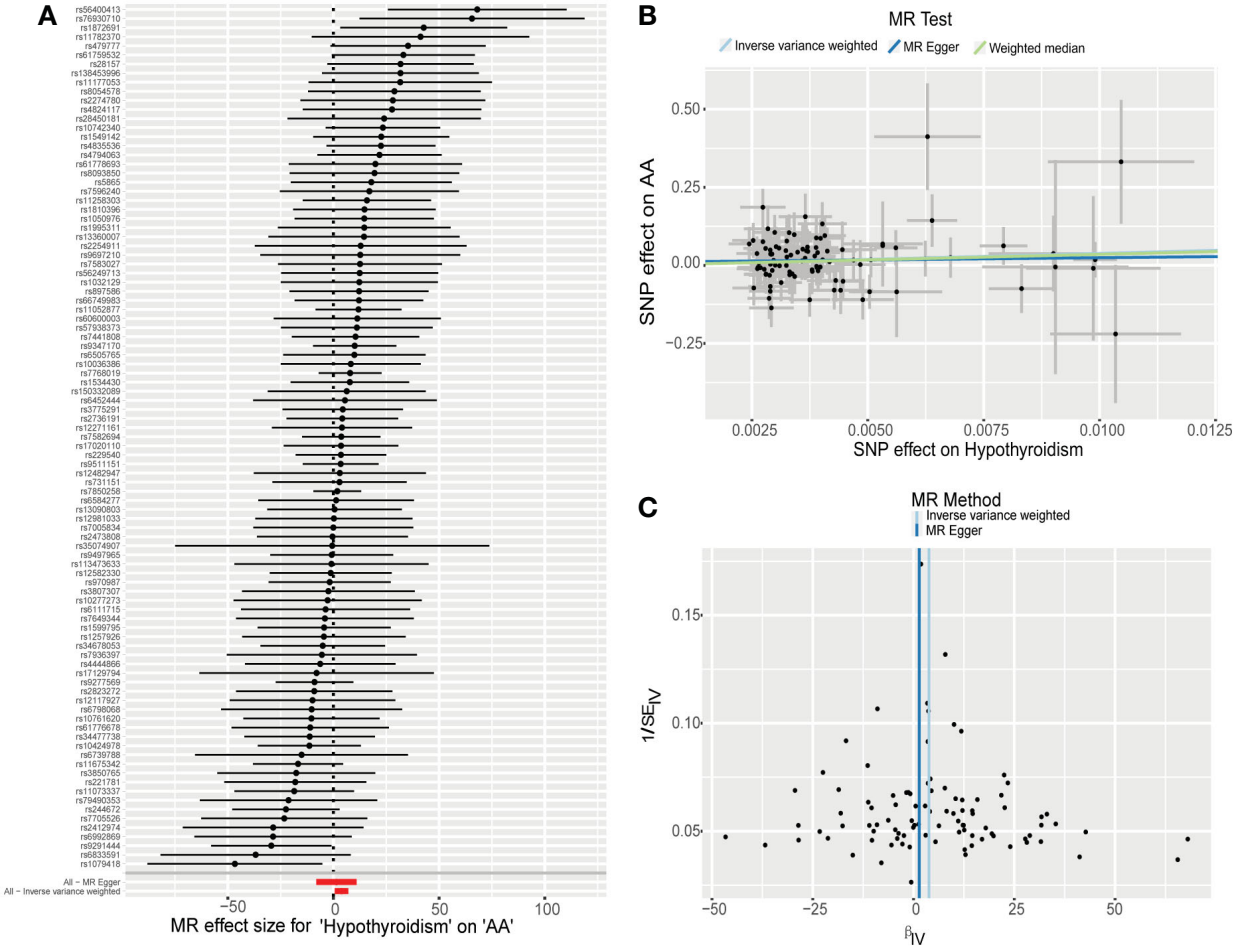
Mendelian randomization (MR) analysis of causal effect of hypothyroidism on alopecia areata (AA). **(A)** The forest plot displays individual estimates of AA risk for each single nucleotide polymorphism (SNP) as black dots, and pooled estimates as red dots. **(B)** The scatter plot illustrates the genetic association between the risk of hypothyroidism on the x-axis and the risk of AA on the y-axis. Each line indicates the causal relationship for each approach. **(C)** Funnel plot shows the overall heterogeneity of MR estimates.

We obtained 95 and 112 independent SNPs as IVs investigating the casual effect of hypothyroidism on AA and AGA, respectively. All the SNPs (with *F* > 10) were deemed strong IVs (**Supplementary Table 2**). Strong evidence of a positive causal effect of hypothyroidism on AA has been demonstrated by both the IVW (OR, 45.6839; 95%CI, 1.8446-1131.4271; *P* = 0.0196; **Table 1**, **Figure 2**) and RAPS (OR, 58.8579; 95%CI, 2.3497-1474.344; *P* = 0.0131; **Table 1**) methods, with MR-Egger and WM estimating in the same direction as the IVW method, despite nonsignificance (**Table 1**, **Figure 1**). Nevertheless, the causal effect of hypothyroidism on AGA was not significantly implicated (**Table 1**, **Supplementary Figure 2**).

No directional pleiotropy, horizontal pleiotropy, or heterogeneity was observed (**Table 2**, **Figure 2**, **Supplementary Figure 2**). None of the single IV were found to drive these causal effects in the leave-one-out analysis (**Supplementary Figures 3C, D**).

## 4 Discussion

Hair loss can have significant psychological effects, impacting the quality of life of those affected negatively. Non-scarring alopecia, including AA and AGA, affect a considerable proportion of the population. This MR study is the first examination of the bidirectional genomic relationship between non-scarring alopecia and hypothyroidism, to our knowledge. Telogen effluvium, another noteworthy subtypes of non-scarring alopecia, was not covered by this study due to the unavailability of GWAS data (30). Our study found genetic support for a causal relationship between AA and hypothyroidism and vice versa, but no such relationship between AGA and hypothyroidism.

The first reported case of AA combined with hypothyroidism was reported by Hurtado et al. in 1960, according to our keyword search in PubMed (31). AA and hypothyroidism can coexist in certain syndromes, such as Laffer Ascher syndrome (32), and autoimmune polyglandular syndrome (33). Several descriptive studies have investigated the correlation between AA and hypothyroidism in the form of prevalence, frequency and clinical significance (7–9, 34–36).

AA is an autoimmune disease that heightens the risk of thyroid problems, particularly hypothyroidism. Thyroid dysfunctions resulting from the autoimmune mechanism are the leading disorders reported in the literature related to AA patients (7, 36–38). A case series of 298 patients with AA in a tertiary referral medical center revealed that hypothyroidism was the most frequent finding of thyroid dysfunction (35). An Egyptian case-control study reported that 16% of AA patients had hypothyroidism, with significant statistical differences in thyroid hormone levels between cases and controls (36). Another case-control study reported a statistically significant high incidence of hypothyroidism (14.1%, *P* = 0.01), with an observed-to-expected ratio of 5.0 (7). AA patients are more prone to subclinical hypothyroidism (OR, 19.61; 95% CI, 4.07-94.41; prevalence, 10.4%), according to a meta-analysis (13). Interestingly, a significant causative effect of hypothyroidism on the increased risk of AA was also identified. A case-control study in the United State using the National AA Registry database consisting of 2,055 self-reported sporadic AA patients and 558 controls investigated the association between history of hypothyroidism and risk of AA (9). A history of hypothyroidism was associated with an increased risk of developing AA after controlling for covariates, including demographics and history of atopic and autoimmune diseases (9).

The present study expands on prior research, revealing a mutual causal relationship between AA and hypothyroidism. The underpinning mechanisms remain obscure, but it is certain that these conditions share a common pathogenesis, including genetic backgrounds and inflammatory processes. Prior studies have demonstrated that human leukocyte antigen (HLA) class II haplotypes susceptible to AA are associated with thyroid autoimmunity (39). HLA-DQB1*03 is positively associated with both autoimmune hypothyroidism and AA (40, 41).

In addition to genetic aspects, autoimmunity also plays a critical role in both AA and hypothyroidism. AA is caused by immune abnormalities in the hair follicles mediated by CD8^+^ cytotoxic T lymphocytes (42). Hashimoto’s thyroiditis, a prevalent autoimmune thyroid disorder, is the leading cause of hypothyroidism in iodine-rich areas (43). A GWAS of AA also identified the genetic basis of non-HLA loci related to cytokines produced by T cells, which are shared with autoimmune hypothyroidism (44). It is assumed that AA and autoimmune hypothyroidism might share mutual circulating inflammatory cytokines (34). Emerging evidence has suggested that an imbalance between regulatory T cells and T helper 17 cells may contribute to the development of AA and thyroid disease (45–47).

AGA is the most prominent form of scalp baldness, impacting 60-70% of the population worldwide (48). Unlike AA, which has an autoimmune background and is closely associated with autoimmune hypothyroidism, AGA is an androgen-dependent trait caused by testosterone and 5α-dihydrotestosterone (48). There are few case-control studies regarding the relationship between AGA and hypothyroidism, with only a few case series reported. Thyroid hormones have a major impact on regulating stem cells in the hair follicle, contributing to the process of hair growth and cycling (49). A thyroxine releasing hormone test performed in 31 female AGA patients showed that 48% of patients tested positive, suggesting that hypothyroidism may interfere with the androgen metabolism and contribute to the clinical presentation of female AGA (50). An observational study found that subclinical hypothyroidism was present in 11% of female patients with diffuse hair loss, including telogen effluvium (78%) and female AGA (22%) (51). Traditionally, alopecia in hypothyroidism has been described as diffuse and secondary to telogen effluvium (52). However, another observational study of 15 female patients with diffuse alopecia secondary to hypothyroidism found no evidence of AGA or telogen effluvium, according to their medical records, clinical assessments, trichoscopic evaluations, and histological analyses (52). In addition, it is worth noting that both alopecia and hypothyroidism are often accompanied by psychiatric comorbidity (18, 53). These findings suggested that hypothyroidism may lead to various types of non-scarring alopecia, beyond AA and AGA, highlighting the intricate and diverse correlation between hypothyroidism and non-scarring alopecia.

Our study has several strengths. First, we conducted bidirectional MR analysis to assess a causal link between non-scarring alopecia and hypothyroidism with minimization of potential confounders. Additionally, we utilized MR-Egger regression intercept and MR-PRESSO tests to identify horizontal pleiotropy. Our study achieved high accuracy with low heterogeneity and bias thanks to the lack of overlap between the GWAS data for AA and hypothyroidism. We also utilized the RAPS method to address issues concerning the bias of weak IVs and to improve the robustness of causal estimates.

However, it has several limitations. First, our study utilized publicly accessible summary statistics obtained from GWAS, which constrained our ability to analyze the effects of other variables, including age, race, and sex. Second, the data for AA and AGA were produced from Finland’s electronic health records, which had a relatively limited sample size. This may explain the lack of accuracy, as evidenced by the wide 95% confidence interval and a low-slope fit line shown in **Figure 2**. Moreover, the misclassification of AA and AGA is also a possibility when employing ICD-codes. Furthermore, we compared specific SNPs with data from the Phenoscanner database to mitigate potential confounding effects. Nonetheless, pleiotropy cannot be completely ruled out since precise biologic function of IVs remains unknown.

## 5 Conclusion

In conclusion, our study demonstrated a bidirectional causal relationship between AA and hypothyroidism. As per our findings, we recommend an integration of thyroid function tests into the clinical assessment of AA patients. Meanwhile, physicians should to be aware that patients with hypothyroidism are at an increased risk of developing AA and should be vigilant in recognizing it. Optimal management of hypothyroidism could potentially reduce the frequency and severity of AA. Similarly, appropriate treatment of AA may decrease the occurrence of hypothyroidism. In addition, although the MR estimate did not provide evidence for a causal association between AGA and hypothyroidism, it does not definitively rule out an association between them. Further research is needed to substantiate these findings.

## Data availability statement

The original contributions presented in the study are included in the article/**Supplementary Materials**, further inquiries can be directed to the corresponding author/s.

## Author contributions

JY: Data curation, Formal Analysis, Investigation, Software, Writing – original draft, Writing – review & editing. ZZ: Conceptualization, Investigation, Methodology, Writing – original draft, Writing – review & editing. CZ: Formal Analysis, Methodology, Software, Writing – original draft, Writing – review & editing. YG: Formal Analysis, Project administration, Writing – original draft, Writing – review & editing. GW: Conceptualization, Project administration, Supervision, Writing – original draft, Writing – review & editing. MF: Conceptualization, Funding acquisition, Project administration, Supervision, Writing – original draft, Writing – review & editing.

## References

[1] Anudeep TC Jeyaraman M Muthu S Rajendran RL Gangadaran P Mishra PC. Advancing regenerative cellular therapies in non-scarring alopecia. Pharmaceutics. (2022) 14:612. doi: 10.3390/pharmaceutics14030612 35335987 PMC8953616

[2] Pratt CH King LE Jr. Messenger AG Christiano AM Sundberg JP. Alopecia areata. Nat Rev Dis Primers. (2017) 3:17011. doi: 10.1038/nrdp.2017.11 28300084 PMC5573125

[3] Spano F Donovan JC. Alopecia areata: Part 1: pathogenesis, diagnosis, and prognosis. Can Fam Physician. (2015) 61:751–5. PMC4569104 26371097

[4] Blume-Peytavi U Blumeyer A Tosti A Finner A Marmol V Trakatelli M. S1 guideline for diagnostic evaluation in androgenetic alopecia in men, women and adolescents. Br J Dermatol. (2011) 164:5–15. doi: 10.1111/j.1365-2133.2010.10011.x 20795997

[5] Jamerson TA Aguh C. An approach to patients with alopecia. Med Clin North Am. (2021) 105:599–610. doi: 10.1016/j.mcna.2021.04.002 34059240

[6] Chiovato L Magri F Carlé A. Hypothyroidism in context: where we’ve been and where we’re going. Adv Ther. (2019) 36:47–58. doi: 10.1007/s12325-019-01080-8 31485975 PMC6822815

[7] Thomas EA Kadyan RS. Alopecia areata and autoimmunity: a clinical study. Indian J Dermatol. (2008) 53:70–4. doi: 10.4103/0019-5154.41650 PMC2763714 19881991

[8] Ghaffari J Rokni GR Kazeminejad A Abedi H. Association among thyroid dysfunction, asthma, allergic rhinitis and eczema in children with alopecia areata. Open Access Maced J Med Sci. (2017) 5:305–9. doi: 10.3889/oamjms.2017.050 PMC5503727 28698747

[9] Barahmani N Schabath MB Duvic M. History of atopy or autoimmunity increases risk of alopecia areata. J Am Acad Dermatol. (2009) 61:581–91. doi: 10.1016/j.jaad.2009.04.031 19608295

[10] Chen CH Wang KH Lin HC Chung SD. Follow-up study on the relationship between alopecia areata and risk of autoimmune diseases. J Dermatol. (2016) 43:228–9. doi: 10.1111/1346-8138.13165 26499292

[11] Han TY Lee JH Noh TK Choi MW Yun JS Lee KH. Alopecia areata and overt thyroid diseases: A nationwide population-based study. J Dermatol. (2018) 45:1411–7. doi: 10.1111/1346-8138.14648 30222206

[12] Kinoshita-Ise M Martinez-Cabriales SA Alhusayen R. Chronological association between alopecia areata and autoimmune thyroid diseases: A systematic review and meta-analysis. J Dermatol. (2019) 46:702–9. doi: 10.1111/1346-8138.14940 31197884

[13] Lee S Lee H Lee CH Lee WS. Comorbidities in alopecia areata: A systematic review and meta-analysis. J Am Acad Dermatol. (2019) 80:466–77.e16. doi: 10.1016/j.jaad.2018.07.013 30031145

[14] Davey Smith G Hemani G. Mendelian randomization: genetic anchors for causal inference in epidemiological studies. Hum Mol Genet. (2014) 23:R89–98. doi: 10.1093/hmg/ddu328 PMC4170722 25064373

[15] Skrivankova VW Richmond RC Woolf BAR Davies NM Swanson SA VanderWeele TJ. Strengthening the reporting of observational studies in epidemiology using mendelian randomisation (STROBE-MR): explanation and elaboration. BMJ. (2021) 375:n2233. doi: 10.1136/bmj.n2233 34702754 PMC8546498

[16] Davies NM Holmes MV Davey Smith G. Reading Mendelian randomisation studies: a guide, glossary, and checklist for clinicians. BMJ. (2018) 362:k601. doi: 10.1136/bmj.k601 30002074 PMC6041728

[17] Sanderson E Glymour MM Holmes MV Kang H Morrison J Munafò MR. Mendelian randomization. Nat Rev Methods Primers. (2022) 2:6. doi: 10.1038/s43586-021-00092-5 37325194 PMC7614635

[18] Huang KP Mullangi S Guo Y Qureshi AA. Autoimmune, atopic, and mental health comorbid conditions associated with alopecia areata in the United States. JAMA Dermatol. (2013) 149:789–94. doi: 10.1001/jamadermatol.2013.3049 23700152

[19] Mohan GC Silverberg JI. Association of vitiligo and alopecia areata with atopic dermatitis: A systematic review and meta-analysis. JAMA Dermatol. (2015) 151:522–8. doi: 10.1001/jamadermatol.2014.3324 25471826

[20] Burgess S Thompson SG. Avoiding bias from weak instruments in Mendelian randomization studies. Int J Epidemiol. (2011) 40:755–64. doi: 10.1093/ije/dyr036 21414999

[21] Bowden J Del Greco MF Minelli C Davey Smith G Sheehan N Thompson J. A framework for the investigation of pleiotropy in two-sample summary data Mendelian randomization. Stat Med. (2017) 36:1783–802. doi: 10.1002/sim.7221 PMC5434863 28114746

[22] Verbanck M Chen CY Neale B Do R. Detection of widespread horizontal pleiotropy in causal relationships inferred from Mendelian randomization between complex traits and diseases. Nat Genet. (2018) 50:693–8. doi: 10.1038/s41588-018-0099-7 PMC6083837 29686387

[23] Bowden J Davey Smith G Burgess S. Mendelian randomization with invalid instruments: effect estimation and bias detection through Egger regression. Int J Epidemiol. (2015) 44:512–25. doi: 10.1093/ije/dyv080 PMC4469799 26050253

[24] Bowden J Davey Smith G Haycock PC Burgess S. Consistent estimation in mendelian randomization with some invalid instruments using a weighted median estimator. Genet Epidemiol. (2016) 40:304–14. doi: 10.1002/gepi.21965 PMC4849733 27061298

[25] Lawlor DA Harbord RM Sterne JA Timpson N Davey Smith G. Mendelian randomization: using genes as instruments for making causal inferences in epidemiology. Stat Med. (2008) 27:1133–63. doi: 10.1002/sim.3034 17886233

[26] Hemani G Bowden J Davey Smith G. Evaluating the potential role of pleiotropy in Mendelian randomization studies. Hum Mol Genet. (2018) 27:R195–r208. doi: 10.1093/hmg/ddy163 29771313 PMC6061876

[27] Bowden J Hemani G Davey Smith G. Invited commentary: detecting individual and global horizontal pleiotropy in mendelian randomization-A job for the humble heterogeneity statistic? Am J Epidemiol. (2018) 187:2681–5. doi: 10.1093/aje/kwy185 PMC6269239 30188969

[28] Bowden J Del Greco MF Minelli C Zhao Q Lawlor DA Sheehan NA. Improving the accuracy of two-sample summary-data Mendelian randomization: moving beyond the NOME assumption. Int J Epidemiol. (2019) 48:728–42. doi: 10.1093/ije/dyy258 PMC6659376 30561657

[29] Chen X Kong J Diao X Cai J Zheng J Xie W. Depression and prostate cancer risk: A Mendelian randomization study. Cancer Med. (2020) 9:9160–7. doi: 10.1002/cam4.3493 PMC7724297 33027558

[30] Alessandrini A Bruni F Piraccini BM Starace M. Common causes of hair loss - clinical manifestations, trichoscopy and therapy. J Eur Acad Dermatol Venereol. (2021) 35:629–40. doi: 10.1111/jdv.17079 33290611

[31] Hurtado OA. [Considerations on a patient of hypothyroidism and pelada]. Rev Chil Pediatr. (1960) 31:192–5. 14405553

[32] Halling F Sandrock D Merten HA Hönig JF. [Ascher’s syndrome]. Dtsch Z Mund Kiefer Gesichtschir. (1991) 15:440–4. 1817784

[33] Sheehan MT Islam R. Silent thyroiditis, isolated corticotropin deficiency, and alopecia universalis in a patient with ulcerative colitis and elevated levels of plasma factor VIII: an unusual case of autoimmune polyglandular syndrome type 3. Endocr Pract. (2009) 15:138–42. doi: 10.4158/ep.15.2.138 19289325

[34] Dai YX Tai YH Chang YT Chen TJ Chen MH. Bidirectional association between alopecia areata and thyroid diseases: a nationwide population-based cohort study. Arch Dermatol Res. (2021) 313:339–46. doi: 10.1007/s00403-020-02109-7 32705333

[35] Patel D Li P Bauer AJ Castelo-Soccio L. Screening guidelines for thyroid function in children with alopecia areata. JAMA Dermatol. (2017) 153:1307–10. doi: 10.1001/jamadermatol.2017.3694 PMC5817442 28973128

[36] Bakry OA Basha MA El Shafiee MK Shehata WA. Thyroid disorders associated with alopecia areata in Egyptian patients. Indian J Dermatol. (2014) 59:49–55. doi: 10.4103/0019-5154.123494 24470660 PMC3884928

[37] Puavilai S Puavilai G Charuwichitratana S Sakuntabhai A Sriprachya-Anunt S. Prevalence of thyroid diseases in patients with alopecia areata. Int J Dermatol. (1994) 33:632–3. doi: 10.1111/j.1365-4362.1994.tb02921.x 8002158

[38] Seyrafi H Akhiani M Abbasi H Mirpour S Gholamrezanezhad A. Evaluation of the profile of alopecia areata and the prevalence of thyroid function test abnormalities and serum autoantibodies in Iranian patients. BMC Dermatol. (2005) 5:11. doi: 10.1186/1471-5945-5-11 16259629 PMC1280924

[39] Noso S Park C Babaya N Hiromine Y Harada T Ito H. Organ specificity in autoimmune diseases: thyroid and islet autoimmunity in alopecia areata. J Clin Endocrinol Metab. (2015) 100:1976–83. doi: 10.1210/jc.2014-3985 25734250

[40] Welsh EA Clark HH Epstein SZ Reveille JD Duvic M. Human leukocyte antigen-DQB1*03 alleles are associated with alopecia areata. J Invest Dermatol. (1994) 103:758–63. doi: 10.1111/1523-1747.ep12412584 7798612

[41] Rekha PL Valluri V Rakh SS Pantula V Ishaq M. Association of HLA DQ B1* and HLA DR B1* alleles with goitrous juvenile autoimmune hypothyroidism–a case control study. J Clin Immunol. (2007) 27:486–9. doi: 10.1007/s10875-007-9102-2 17588142

[42] Rajabi F Drake LA Senna MM Rezaei N. Alopecia areata: a review of disease pathogenesis. Br J Dermatol. (2018) 179:1033–48. doi: 10.1111/bjd.16808 29791718

[43] Lee HJ Stefan-Lifshitz M Li CW Tomer Y. Genetics and epigenetics of autoimmune thyroid diseases: Translational implications. Best Pract Res Clin Endocrinol Metab. (2023) 37:101661. doi: 10.1016/j.beem.2022.101661 35459628 PMC9550878

[44] Petukhova L Duvic M Hordinsky M Norris D Price V Shimomura Y. Genome-wide association study in alopecia areata implicates both innate and adaptive immunity. Nature. (2010) 466:113–7. doi: 10.1038/nature09114 PMC2921172 20596022

[45] Dejaco C Duftner C Grubeck-Loebenstein B Schirmer M. Imbalance of regulatory T cells in human autoimmune diseases. Immunology. (2006) 117:289–300. doi: 10.1111/j.1365-2567.2005.02317.x 16476048 PMC1782226

[46] Marazuela M García-López MA Figueroa-Vega N de la Fuente H Alvarado-Sánchez B Monsiváis-Urenda A. Regulatory T cells in human autoimmune thyroid disease. J Clin Endocrinol Metab. (2006) 91:3639–46. doi: 10.1210/jc.2005-2337 16804051

[47] Han YM Sheng YY Xu F Qi SS Liu XJ Hu RM. Imbalance of T-helper 17 and regulatory T cells in patients with alopecia areata. J Dermatol. (2015) 42:981–8. doi: 10.1111/1346-8138.12978 26077574

[48] Jain R De-Eknamkul W. Potential targets in the discovery of new hair growth promoters for androgenic alopecia. Expert Opin Ther Targets. (2014) 18:787–806. doi: 10.1517/14728222.2014.922956 24873677

[49] Contreras-Jurado C Lorz C García-Serrano L Paramio JM Aranda A. Thyroid hormone signaling controls hair follicle stem cell function. Mol Biol Cell. (2015) 26:1263–72. doi: 10.1091/mbc.E14-07-1251 PMC4454174 25657324

[50] Schmidt JB Schurz B Huber J Spona J. [Hypothyroidism and hyperprolactinemia as a possible cause of androgenetic alopecia in the female]. Z Hautkr. (1989) 64:9–12. 2494810

[51] Poonia K Thami GP Bhalla M Jaiswal S Sandhu J. NonScarring Diffuse Hair Loss in Women: a Clinico-Etiological Study from tertiary care center in North-West India. J Cosmet Dermatol. (2019) 18:401–7. doi: 10.1111/jocd.12559 29774652

[52] Leal-Osuna SE Becerril-Parra DE Tinoco-Fragoso F García-Gil AB Vega-Memije ME Lammoglia-Ordiales L. Clinical, trichoscopic, and histopathologic characteristics of patients with alopecia and hypothyroidism: An observational study. J Am Acad Dermatol. (2018) 79:958–60. doi: 10.1016/j.jaad.2018.04.050 29746875

[53] Siegmann EM Müller HHO Luecke C Philipsen A Kornhuber J Grömer TW. Association of depression and anxiety disorders with autoimmune thyroiditis: A systematic review and meta-analysis. JAMA Psychiatry. (2018) 75:577–84. doi: 10.1001/jamapsychiatry.2018.0190 PMC6137529 29800939

