# Supplementary material for: Association between non-scarring alopecia and hypothyroidism: a bidirectional two-sample Mendelian randomization study
Source: Front Endocrinol (Lausanne). 2024 Mar 18;15:1356832. doi: 10.3389/fendo.2024.1356832 (PMC10982309; doi:10.3389/fendo.2024.1356832)
Supplement: Supplementary file 1 [file DataSheet_1.docx]

Supplementary Material

## 1 Supplementary Figures


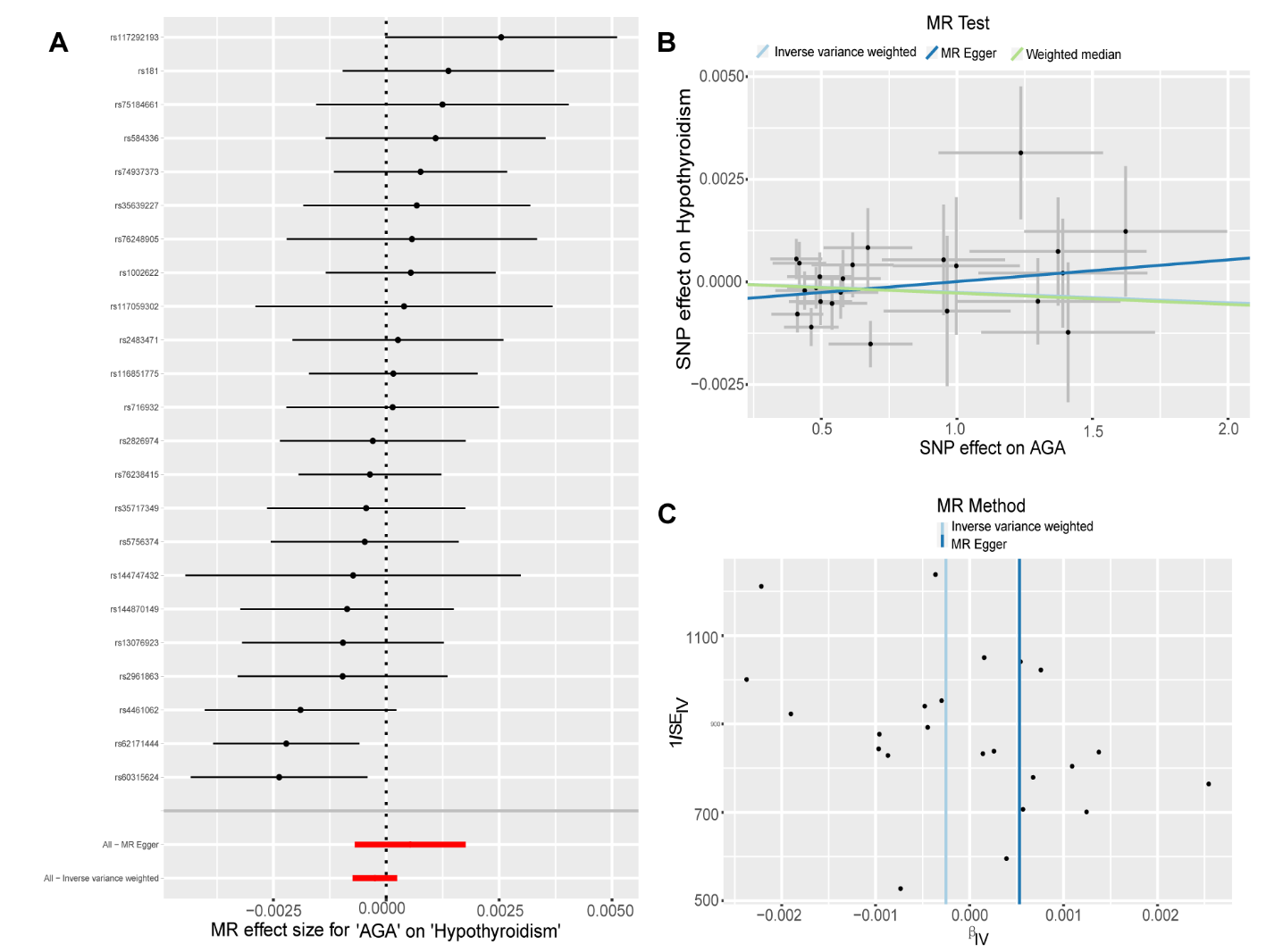


**Supplementary Figure 1.** Mendelian randomization (MR) analysis of causal effect of androgenetic alopecia (AGA) on hypothyroidism. (A)The forest plot displays individual estimates of hypothyroidism risk for each single nucleotide polymorphism (SNP) as black dots, and pooled estimates as red dots. (B) The scatter plot illustrates the genetic association between the risk of AGA on the x-axis and the risk of hypothyroidism on the y-axis. Each line indicates the causal relationship for each approach. (C)Funnel plot shows the overall heterogeneity of MR estimates.


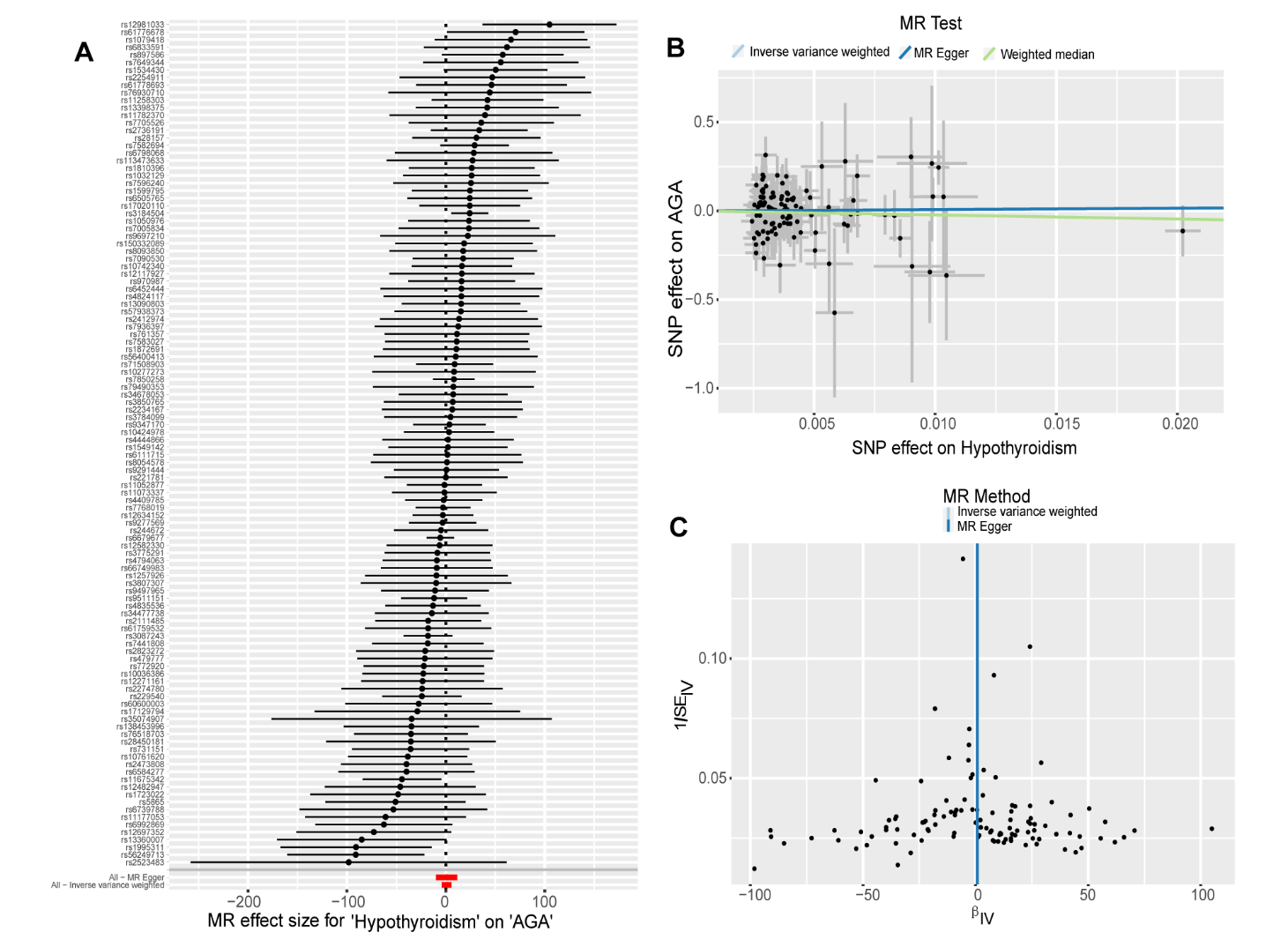


**Supplementary Figure 2.** Mendelian randomization (MR) analysis of causal effect of hypothyroidism on androgenetic alopecia (AGA). (A)The forest plot displays individual estimates of AGA risk for each single nucleotide polymorphism (SNP) as black dots, and pooled estimates as red dots. (B) The scatter plot illustrates the genetic association between the risk of hypothyroidism on the x-axis and the risk of AGA on the y-axis. Each line indicates the causal relationship for each approach. (C)Funnel plot shows the overall heterogeneity of MR estimates.


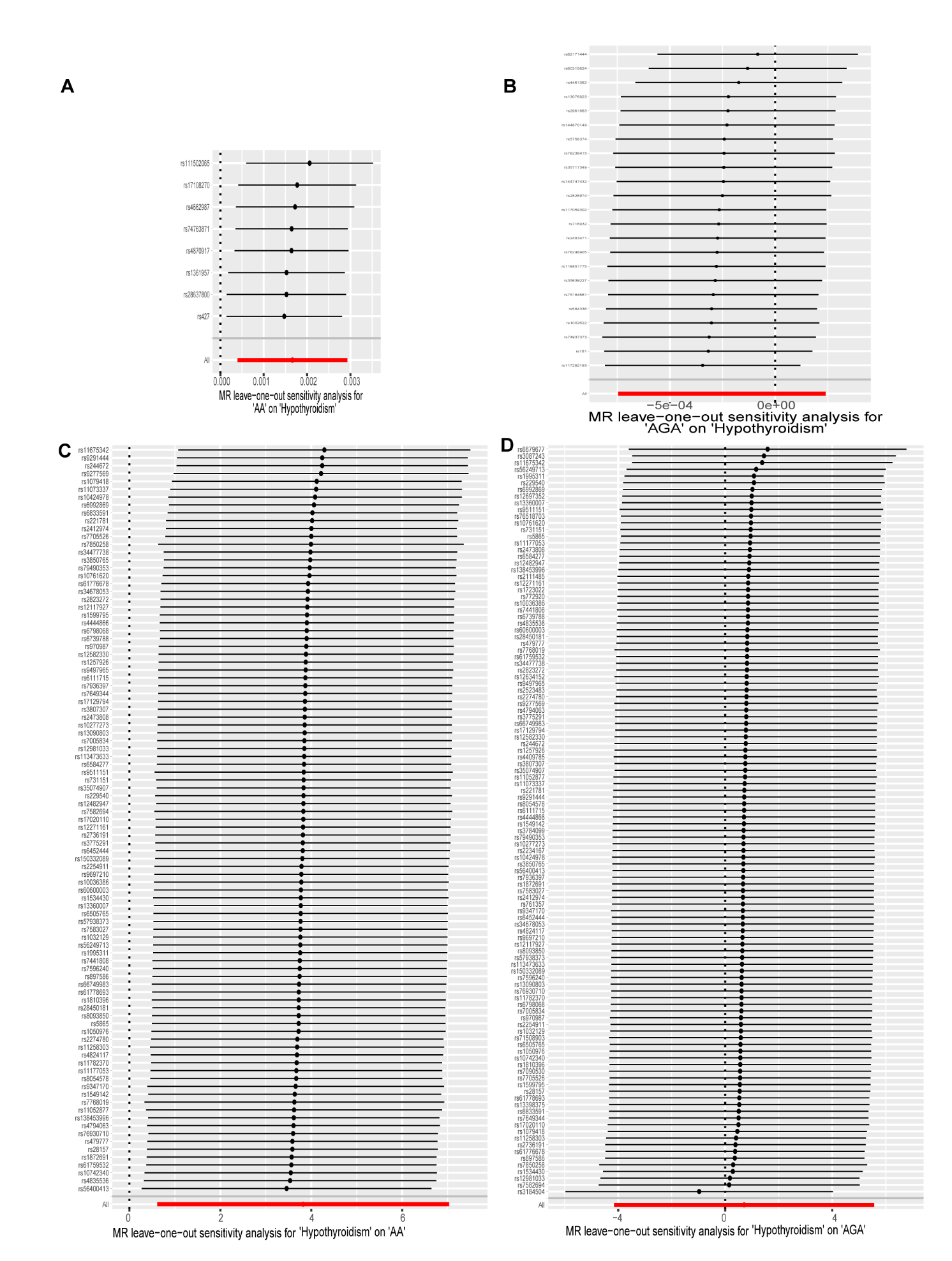


**Supplementary Figure 3.** The Forest plot displays the leave-one-out analysis of the association between non-scarring alopecia and hypothyroidism, with separate panels for (A) alopecia areata (AA) and the risk of hypothyroidism, (B) androgenetic alopecia (AGA) and the risk of hypothyroidism, (C) hypothyroidism and the risk of AA, and (D) hypothyroidism and the risk of AGA. Each dot represents the Mendelian randomization (MR) effect size after excluding the individual single nucleotide polymorphism (SNP) on the left side.

## 2 Supplementary Tables

**TABLE S1** **Details of the** **GWAS datasets**

| Trait | GWAS ID | Year | Cases | Controls | Sex | Ancestry |
| --- | --- | --- | --- | --- | --- | --- |
| AA | finngen-L12_ALOPECAREATA | 2022 | 682 | 361,140 | Male and Female | European |
| AGA | finngen_R9_L12_ALOPECANDRO | 2022 | 195 | 201,019 | Male and Female | European |
| Hypo | ukb-b-19732 | 2018 | 22,687 | 440,246 | Male and Female | European |

GWAS, genome-wide association study; AA, alopecia areata; AGA, androgenetic alopecia; Hypo, hypothyroidism; UKB, UK Biobank.

**TABLE S2 Final SNPs used as genetic instruments for each trait**

| Causal effect | SNP | EA | OA | β | SE | *P* | *F* |
| --- | --- | --- | --- | --- | --- | --- | --- |
| AA on Hypo | rs111502065 | A | G | -1.21E+00 | 2.69E-01 | 7.11E-06 | 20.16 |
|  | rs1361957 | G | A | -2.37E-01 | 5.37E-02 | 9.94E-06 | 19.52 |
|  | rs17108270 | A | T | -2.94E-01 | 6.35E-02 | 3.80E-06 | 21.36 |
|  | rs28637800 | A | G | 4.37E-01 | 9.36E-02 | 3.12E-06 | 21.74 |
|  | rs427 | A | G | 2.80E-01 | 5.95E-02 | 2.58E-06 | 22.10 |
|  | rs4662987 | C | T | 5.31E-01 | 1.20E-01 | 9.20E-06 | 19.67 |
|  | rs4870917 | C | G | 3.97E-01 | 8.97E-02 | 9.75E-06 | 19.56 |
|  | rs74763871 | T | C | 5.21E-01 | 1.15E-01 | 5.90E-06 | 20.52 |
| AGA on Hypo | rs1002622 | T | C | 1.37E+00 | 3.26E-01 | 2.57E-05 | 17.71 |
|  | rs116851775 | T | C | 1.39E+00 | 3.12E-01 | 8.24E-06 | 19.88 |
|  | rs117059302 | G | A | 9.98E-01 | 2.34E-01 | 2.03E-05 | 18.16 |
|  | rs117292193 | C | T | 1.24E+00 | 3.04E-01 | 4.69E-05 | 16.57 |
|  | rs13076923 | G | A | -4.97E-01 | 1.16E-01 | 1.91E-05 | 18.27 |
|  | rs144747432 | T | C | 9.64E-01 | 2.34E-01 | 3.80E-05 | 16.97 |
|  | rs144870149 | A | G | 1.41E+00 | 3.21E-01 | 1.10E-05 | 19.34 |
|  | rs181 | G | A | 4.07E-01 | 9.58E-02 | 2.11E-05 | 18.08 |
|  | rs2483471 | A | G | -4.94E-01 | 1.21E-01 | 4.18E-05 | 16.79 |
|  | rs2826974 | G | A | 4.80E-01 | 1.07E-01 | 7.15E-06 | 20.15 |
|  | rs2961863 | C | T | -5.39E-01 | 1.29E-01 | 3.10E-05 | 17.35 |
|  | rs35639227 | T | C | 6.15E-01 | 1.51E-01 | 4.85E-05 | 16.51 |
|  | rs35717349 | T | C | 5.71E-01 | 1.38E-01 | 3.55E-05 | 17.10 |
|  | rs4461062 | T | C | 4.11E-01 | 9.54E-02 | 1.61E-05 | 18.60 |
|  | rs5756374 | T | C | 4.38E-01 | 1.08E-01 | 4.92E-05 | 16.48 |
|  | rs584336 | A | G | 4.19E-01 | 9.85E-02 | 2.12E-05 | 18.08 |
|  | rs60315624 | T | C | -4.63E-01 | 1.00E-01 | 3.81E-06 | 21.36 |
|  | rs62171444 | C | A | -6.81E-01 | 1.54E-01 | 1.01E-05 | 19.50 |
|  | rs716932 | A | C | 5.79E-01 | 1.39E-01 | 2.98E-05 | 17.43 |
|  | rs74937373 | A | G | 1.62E+00 | 3.74E-01 | 1.43E-05 | 18.83 |
|  | rs75184661 | T | C | 6.71E-01 | 1.64E-01 | 4.03E-05 | 16.86 |
|  | rs76238415 | G | A | -1.30E+00 | 3.05E-01 | 2.07E-05 | 18.12 |
|  | rs76248905 | C | T | 9.51E-01 | 2.27E-01 | 2.85E-05 | 17.51 |
| Hypo on AA | rs10036386 | T | C | 3.25E-03 | 4.56E-04 | 9.70E-13 | 50.91 |
|  | rs10277273 | G | T | -2.68E-03 | 4.53E-04 | 3.10E-09 | 35.14 |
|  | rs1032129 | C | A | -2.92E-03 | 4.64E-04 | 3.20E-10 | 39.53 |
|  | rs10424978 | A | C | -4.47E-03 | 4.57E-04 | 1.60E-22 | 95.38 |
|  | rs1050976 | T | C | 3.23E-03 | 4.43E-04 | 2.90E-13 | 53.31 |
|  | rs10742340 | C | T | 4.07E-03 | 4.57E-04 | 4.60E-19 | 79.58 |
|  | rs10761620 | G | A | -3.42E-03 | 4.47E-04 | 1.90E-14 | 58.59 |
|  | rs1079418 | G | A | -2.92E-03 | 4.80E-04 | 1.20E-09 | 37.01 |
|  | rs11052877 | G | A | -5.32E-03 | 4.59E-04 | 5.00E-31 | 134.18 |
|  | rs11073337 | C | A | 4.28E-03 | 5.12E-04 | 6.50E-17 | 69.82 |
|  | rs11177053 | C | T | -2.53E-03 | 4.51E-04 | 2.10E-08 | 31.44 |
|  | rs11258303 | A | C | 3.70E-03 | 5.10E-04 | 4.20E-13 | 52.56 |
|  | rs113473633 | G | A | -9.86E-03 | 1.45E-03 | 1.00E-11 | 46.25 |
|  | rs11675342 | T | C | 5.04E-03 | 4.49E-04 | 2.70E-29 | 126.24 |
|  | rs11782370 | T | C | -2.84E-03 | 5.20E-04 | 4.40E-08 | 29.95 |
|  | rs12117927 | A | C | 2.80E-03 | 4.54E-04 | 6.70E-10 | 38.10 |
|  | rs12271161 | A | G | -3.58E-03 | 5.61E-04 | 1.70E-10 | 40.73 |
|  | rs12482947 | C | T | 2.65E-03 | 4.55E-04 | 5.50E-09 | 33.99 |
|  | rs1257926 | A | G | 2.77E-03 | 4.44E-04 | 4.80E-10 | 38.78 |
|  | rs12582330 | T | G | -3.97E-03 | 4.99E-04 | 1.60E-15 | 63.48 |
|  | rs12981033 | G | A | -3.00E-03 | 4.54E-04 | 3.80E-11 | 43.72 |
|  | rs13090803 | T | G | 4.84E-03 | 5.46E-04 | 7.50E-19 | 78.62 |
|  | rs13360007 | G | A | 3.60E-03 | 6.47E-04 | 2.70E-08 | 30.92 |
|  | rs138453996 | A | G | 1.05E-02 | 1.58E-03 | 3.40E-11 | 43.94 |
|  | rs150332089 | A | C | 3.65E-03 | 6.46E-04 | 1.70E-08 | 31.87 |
|  | rs1534430 | T | C | -3.86E-03 | 4.55E-04 | 2.20E-17 | 71.94 |
|  | rs1549142 | T | C | 3.89E-03 | 5.30E-04 | 2.10E-13 | 53.89 |
|  | rs1599795 | A | T | 3.93E-03 | 5.62E-04 | 2.80E-12 | 48.80 |
|  | rs17020110 | C | T | 4.69E-03 | 5.02E-04 | 9.20E-21 | 87.33 |
|  | rs17129794 | C | A | 3.37E-03 | 5.88E-04 | 1.00E-08 | 32.79 |
|  | rs1810396 | G | A | -3.88E-03 | 4.78E-04 | 4.70E-16 | 65.91 |
|  | rs1872691 | A | G | -3.65E-03 | 5.75E-04 | 2.10E-10 | 40.38 |
|  | rs1995311 | G | T | -2.61E-03 | 4.43E-04 | 4.00E-09 | 34.60 |
|  | rs221781 | G | A | 4.41E-03 | 6.99E-04 | 2.70E-10 | 39.91 |
|  | rs2254911 | C | T | -5.32E-03 | 8.67E-04 | 8.50E-10 | 37.64 |
|  | rs2274780 | C | T | 2.44E-03 | 4.44E-04 | 3.70E-08 | 30.31 |
|  | rs229540 | G | T | 5.07E-03 | 4.48E-04 | 1.30E-29 | 127.76 |
|  | rs2412974 | T | C | -2.54E-03 | 4.61E-04 | 3.70E-08 | 30.32 |
|  | rs244672 | T | C | -4.89E-03 | 6.70E-04 | 2.90E-13 | 53.30 |
|  | rs2473808 | C | T | -3.10E-03 | 4.70E-04 | 4.40E-11 | 43.41 |
|  | rs2736191 | G | C | -9.00E-03 | 1.39E-03 | 1.10E-10 | 41.65 |
|  | rs28157 | T | G | -3.30E-03 | 4.78E-04 | 4.70E-12 | 47.80 |
|  | rs2823272 | A | T | -3.31E-03 | 4.78E-04 | 4.00E-12 | 48.10 |
|  | rs28450181 | G | A | 3.25E-03 | 5.51E-04 | 3.50E-09 | 34.87 |
|  | rs34477738 | G | A | 4.31E-03 | 6.22E-04 | 4.30E-12 | 48.00 |
|  | rs34678053 | A | G | -3.80E-03 | 5.03E-04 | 4.10E-14 | 57.12 |
|  | rs35074907 | A | G | 9.05E-03 | 1.58E-03 | 1.00E-08 | 32.81 |
|  | rs3775291 | T | C | -4.03E-03 | 4.84E-04 | 9.20E-17 | 69.13 |
|  | rs3807307 | C | T | 2.63E-03 | 4.44E-04 | 3.00E-09 | 35.18 |
|  | rs3850765 | C | T | 3.13E-03 | 4.50E-04 | 3.30E-12 | 48.48 |
|  | rs4444866 | T | C | -3.31E-03 | 4.98E-04 | 2.90E-11 | 44.23 |
|  | rs4794063 | T | C | 3.98E-03 | 5.07E-04 | 3.90E-15 | 61.77 |
|  | rs479777 | C | T | -2.99E-03 | 4.68E-04 | 1.60E-10 | 40.93 |
|  | rs4824117 | G | A | -2.74E-03 | 4.71E-04 | 6.10E-09 | 33.80 |
|  | rs4835536 | T | G | -6.39E-03 | 5.42E-04 | 4.30E-32 | 139.03 |
|  | rs56249713 | C | T | -2.93E-03 | 4.52E-04 | 8.20E-11 | 42.21 |
|  | rs56400413 | A | T | 2.74E-03 | 4.90E-04 | 2.30E-08 | 31.19 |
|  | rs57938373 | T | C | 4.13E-03 | 6.25E-04 | 4.00E-11 | 43.61 |
|  | rs5865 | T | C | -3.09E-03 | 4.69E-04 | 4.80E-11 | 43.25 |
|  | rs60600003 | G | T | 4.45E-03 | 7.42E-04 | 2.00E-09 | 35.98 |
|  | rs6111715 | C | G | -3.80E-03 | 5.77E-04 | 4.80E-11 | 43.25 |
|  | rs61759532 | T | C | 4.02E-03 | 5.28E-04 | 2.70E-14 | 57.92 |
|  | rs61776678 | A | G | -2.88E-03 | 4.50E-04 | 1.60E-10 | 40.91 |
|  | rs61778693 | T | G | -3.02E-03 | 5.14E-04 | 4.20E-09 | 34.53 |
|  | rs6452444 | C | T | 2.97E-03 | 5.19E-04 | 1.10E-08 | 32.66 |
|  | rs6505765 | G | C | 3.34E-03 | 4.67E-04 | 8.00E-13 | 51.28 |
|  | rs6584277 | G | A | -2.90E-03 | 4.43E-04 | 6.20E-11 | 42.77 |
|  | rs66749983 | T | A | 3.71E-03 | 4.80E-04 | 1.00E-14 | 59.82 |
|  | rs6739788 | T | A | 5.62E-03 | 9.81E-04 | 9.90E-09 | 32.87 |
|  | rs6798068 | A | G | 2.80E-03 | 4.73E-04 | 3.20E-09 | 35.03 |
|  | rs6833591 | G | A | -2.87E-03 | 4.66E-04 | 7.30E-10 | 37.93 |
|  | rs6992869 | C | T | 2.90E-03 | 4.58E-04 | 2.50E-10 | 40.02 |
|  | rs7005834 | T | C | -3.24E-03 | 4.79E-04 | 1.20E-11 | 45.93 |
|  | rs731151 | A | G | 4.17E-03 | 5.96E-04 | 2.40E-12 | 49.11 |
|  | rs7441808 | G | A | 3.92E-03 | 4.83E-04 | 4.80E-16 | 65.87 |
|  | rs7582694 | G | C | -6.78E-03 | 5.30E-04 | 1.60E-37 | 163.91 |
|  | rs7583027 | C | A | 2.90E-03 | 4.62E-04 | 3.30E-10 | 39.47 |
|  | rs7596240 | G | A | 2.90E-03 | 4.98E-04 | 6.10E-09 | 33.79 |
|  | rs7649344 | C | T | -2.62E-03 | 4.45E-04 | 4.00E-09 | 34.60 |
|  | rs76930710 | C | T | -6.29E-03 | 1.15E-03 | 4.20E-08 | 30.06 |
|  | rs7705526 | A | C | -2.90E-03 | 4.78E-04 | 1.40E-09 | 36.68 |
|  | rs7768019 | G | C | -7.93E-03 | 5.14E-04 | 1.20E-53 | 237.76 |
|  | rs7850258 | G | A | 9.90E-03 | 4.70E-04 | 1.90E-98 | 443.46 |
|  | rs7936397 | A | G | -2.94E-03 | 4.98E-04 | 3.60E-09 | 34.84 |
|  | rs79490353 | C | T | 1.03E-02 | 1.41E-03 | 2.30E-13 | 53.77 |
|  | rs8054578 | G | A | -3.41E-03 | 5.30E-04 | 1.20E-10 | 41.39 |
|  | rs8093850 | G | A | 2.79E-03 | 4.78E-04 | 4.90E-09 | 34.24 |
|  | rs897586 | A | G | -3.49E-03 | 4.63E-04 | 5.10E-14 | 56.71 |
|  | rs9277569 | T | C | 8.32E-03 | 7.08E-04 | 7.60E-32 | 137.93 |
|  | rs9291444 | T | C | 3.75E-03 | 4.44E-04 | 3.30E-17 | 71.15 |
|  | rs9347170 | T | C | -5.60E-03 | 4.68E-04 | 4.30E-33 | 143.60 |
|  | rs9497965 | T | C | 3.89E-03 | 4.51E-04 | 5.80E-18 | 74.59 |
|  | rs9511151 | A | G | -6.24E-03 | 4.67E-04 | 1.10E-40 | 178.44 |
|  | rs9697210 | A | G | -3.68E-03 | 6.29E-04 | 5.00E-09 | 34.20 |
|  | rs970987 | A | C | -3.90E-03 | 4.70E-04 | 1.00E-16 | 68.96 |
| Hypo on AGA | rs10036386 | T | C | 3.25E-03 | 4.56E-04 | 9.70E-13 | 50.91 |
|  | rs10277273 | G | T | -2.68E-03 | 4.53E-04 | 3.10E-09 | 35.14 |
|  | rs1032129 | C | A | -2.92E-03 | 4.64E-04 | 3.20E-10 | 39.53 |
|  | rs10424978 | A | C | -4.47E-03 | 4.57E-04 | 1.60E-22 | 95.38 |
|  | rs1050976 | T | C | 3.23E-03 | 4.43E-04 | 2.90E-13 | 53.31 |
|  | rs10742340 | C | T | 4.07E-03 | 4.57E-04 | 4.60E-19 | 79.58 |
|  | rs10761620 | G | A | -3.42E-03 | 4.47E-04 | 1.90E-14 | 58.59 |
|  | rs1079418 | G | A | -2.92E-03 | 4.80E-04 | 1.20E-09 | 37.01 |
|  | rs11052877 | G | A | -5.32E-03 | 4.59E-04 | 5.00E-31 | 134.18 |
|  | rs11073337 | C | A | 4.28E-03 | 5.12E-04 | 6.50E-17 | 69.82 |
|  | rs11177053 | C | T | -2.53E-03 | 4.51E-04 | 2.10E-08 | 31.44 |
|  | rs11258303 | A | C | 3.70E-03 | 5.10E-04 | 4.20E-13 | 52.56 |
|  | rs113473633 | G | A | -9.86E-03 | 1.45E-03 | 1.00E-11 | 46.25 |
|  | rs11675342 | T | C | 5.04E-03 | 4.49E-04 | 2.70E-29 | 126.24 |
|  | rs11782370 | T | C | -2.84E-03 | 5.20E-04 | 4.40E-08 | 29.95 |
|  | rs12117927 | A | C | 2.80E-03 | 4.54E-04 | 6.70E-10 | 38.10 |
|  | rs12271161 | A | G | -3.58E-03 | 5.61E-04 | 1.70E-10 | 40.73 |
|  | rs12482947 | C | T | 2.65E-03 | 4.55E-04 | 5.50E-09 | 33.99 |
|  | rs1257926 | A | G | 2.77E-03 | 4.44E-04 | 4.80E-10 | 38.78 |
|  | rs12582330 | T | G | -3.97E-03 | 4.99E-04 | 1.60E-15 | 63.48 |
|  | rs12634152 | T | C | -6.51E-03 | 4.46E-04 | 2.40E-48 | 213.51 |
|  | rs12697352 | A | G | -2.60E-03 | 4.68E-04 | 2.60E-08 | 30.98 |
|  | rs12981033 | G | A | -3.00E-03 | 4.54E-04 | 3.80E-11 | 43.72 |
|  | rs13090803 | T | G | 4.84E-03 | 5.46E-04 | 7.50E-19 | 78.62 |
|  | rs13360007 | G | A | 3.60E-03 | 6.47E-04 | 2.70E-08 | 30.92 |
|  | rs13398375 | C | T | -2.90E-03 | 4.91E-04 | 3.50E-09 | 34.87 |
|  | rs138453996 | A | G | 1.05E-02 | 1.58E-03 | 3.40E-11 | 43.94 |
|  | rs150332089 | A | C | 3.65E-03 | 6.46E-04 | 1.70E-08 | 31.87 |
|  | rs1534430 | T | C | -3.86E-03 | 4.55E-04 | 2.20E-17 | 71.94 |
|  | rs1549142 | T | C | 3.89E-03 | 5.30E-04 | 2.10E-13 | 53.89 |
|  | rs1599795 | A | T | 3.93E-03 | 5.62E-04 | 2.80E-12 | 48.80 |
|  | rs17020110 | C | T | 4.69E-03 | 5.02E-04 | 9.20E-21 | 87.33 |
|  | rs17129794 | C | A | 3.37E-03 | 5.88E-04 | 1.00E-08 | 32.79 |
|  | rs1723022 | T | G | 2.73E-03 | 4.62E-04 | 3.50E-09 | 34.86 |
|  | rs1810396 | G | A | -3.88E-03 | 4.78E-04 | 4.70E-16 | 65.91 |
|  | rs1872691 | A | G | -3.65E-03 | 5.75E-04 | 2.10E-10 | 40.38 |
|  | rs1995311 | G | T | -2.61E-03 | 4.43E-04 | 4.00E-09 | 34.60 |
|  | rs2111485 | G | A | 3.76E-03 | 4.53E-04 | 9.20E-17 | 69.14 |
|  | rs221781 | G | A | 4.41E-03 | 6.99E-04 | 2.70E-10 | 39.91 |
|  | rs2234167 | A | G | 4.25E-03 | 6.50E-04 | 5.90E-11 | 42.84 |
|  | rs2254911 | C | T | -5.32E-03 | 8.67E-04 | 8.50E-10 | 37.64 |
|  | rs2274780 | C | T | 2.44E-03 | 4.44E-04 | 3.70E-08 | 30.31 |
|  | rs229540 | G | T | 5.07E-03 | 4.48E-04 | 1.30E-29 | 127.76 |
|  | rs2412974 | T | C | -2.54E-03 | 4.61E-04 | 3.70E-08 | 30.32 |
|  | rs244672 | T | C | -4.89E-03 | 6.70E-04 | 2.90E-13 | 53.30 |
|  | rs2473808 | C | T | -3.10E-03 | 4.70E-04 | 4.40E-11 | 43.41 |
|  | rs2523483 | G | T | -5.85E-03 | 7.82E-04 | 7.70E-14 | 55.87 |
|  | rs2736191 | G | C | -9.00E-03 | 1.39E-03 | 1.10E-10 | 41.65 |
|  | rs28157 | T | G | -3.30E-03 | 4.78E-04 | 4.70E-12 | 47.80 |
|  | rs2823272 | A | T | -3.31E-03 | 4.78E-04 | 4.00E-12 | 48.10 |
|  | rs28450181 | G | A | 3.25E-03 | 5.51E-04 | 3.50E-09 | 34.87 |
|  | rs3087243 | A | G | -8.56E-03 | 4.45E-04 | 1.70E-82 | 370.14 |
|  | rs3184504 | C | T | -1.01E-02 | 4.42E-04 | 3.00E-116 | 525.30 |
|  | rs34477738 | G | A | 4.31E-03 | 6.22E-04 | 4.30E-12 | 48.00 |
|  | rs34678053 | A | G | -3.80E-03 | 5.03E-04 | 4.10E-14 | 57.12 |
|  | rs35074907 | A | G | 9.05E-03 | 1.58E-03 | 1.00E-08 | 32.81 |
|  | rs3775291 | T | C | -4.03E-03 | 4.84E-04 | 9.20E-17 | 69.13 |
|  | rs3784099 | A | G | -3.22E-03 | 4.92E-04 | 5.90E-11 | 42.86 |
|  | rs3807307 | C | T | 2.63E-03 | 4.44E-04 | 3.00E-09 | 35.18 |
|  | rs3850765 | C | T | 3.13E-03 | 4.50E-04 | 3.30E-12 | 48.48 |
|  | rs4409785 | C | T | 6.80E-03 | 5.86E-04 | 4.30E-31 | 134.47 |
|  | rs4444866 | T | C | -3.31E-03 | 4.98E-04 | 2.90E-11 | 44.23 |
|  | rs4794063 | T | C | 3.98E-03 | 5.07E-04 | 3.90E-15 | 61.77 |
|  | rs479777 | C | T | -2.99E-03 | 4.68E-04 | 1.60E-10 | 40.93 |
|  | rs4824117 | G | A | -2.74E-03 | 4.71E-04 | 6.10E-09 | 33.80 |
|  | rs4835536 | T | G | -6.39E-03 | 5.42E-04 | 4.30E-32 | 139.03 |
|  | rs56249713 | C | T | -2.93E-03 | 4.52E-04 | 8.20E-11 | 42.21 |
|  | rs56400413 | A | T | 2.74E-03 | 4.90E-04 | 2.30E-08 | 31.19 |
|  | rs57938373 | T | C | 4.13E-03 | 6.25E-04 | 4.00E-11 | 43.61 |
|  | rs5865 | T | C | -3.09E-03 | 4.69E-04 | 4.80E-11 | 43.25 |
|  | rs60600003 | G | T | 4.45E-03 | 7.42E-04 | 2.00E-09 | 35.98 |
|  | rs6111715 | C | G | -3.80E-03 | 5.77E-04 | 4.80E-11 | 43.25 |
|  | rs61759532 | T | C | 4.02E-03 | 5.28E-04 | 2.70E-14 | 57.92 |
|  | rs61776678 | A | G | -2.88E-03 | 4.50E-04 | 1.60E-10 | 40.91 |
|  | rs61778693 | T | G | -3.02E-03 | 5.14E-04 | 4.20E-09 | 34.53 |
|  | rs6452444 | C | T | 2.97E-03 | 5.19E-04 | 1.10E-08 | 32.66 |
|  | rs6505765 | G | C | 3.34E-03 | 4.67E-04 | 8.00E-13 | 51.28 |
|  | rs6584277 | G | A | -2.90E-03 | 4.43E-04 | 6.20E-11 | 42.77 |
|  | rs66749983 | T | A | 3.71E-03 | 4.80E-04 | 1.00E-14 | 59.82 |
|  | rs6679677 | A | C | 2.02E-02 | 7.34E-04 | 5.11E-167 | 758.72 |
|  | rs6739788 | T | A | 5.62E-03 | 9.81E-04 | 9.90E-09 | 32.87 |
|  | rs6798068 | A | G | 2.80E-03 | 4.73E-04 | 3.20E-09 | 35.03 |
|  | rs6833591 | G | A | -2.87E-03 | 4.66E-04 | 7.30E-10 | 37.93 |
|  | rs6992869 | C | T | 2.90E-03 | 4.58E-04 | 2.50E-10 | 40.02 |
|  | rs7005834 | T | C | -3.24E-03 | 4.79E-04 | 1.20E-11 | 45.93 |
|  | rs7090530 | A | C | 4.16E-03 | 4.52E-04 | 3.70E-20 | 84.58 |
|  | rs71508903 | T | C | 6.62E-03 | 5.65E-04 | 1.00E-31 | 137.36 |
|  | rs731151 | A | G | 4.17E-03 | 5.96E-04 | 2.40E-12 | 49.11 |
|  | rs7441808 | G | A | 3.92E-03 | 4.83E-04 | 4.80E-16 | 65.87 |
|  | rs7582694 | G | C | -6.78E-03 | 5.30E-04 | 1.60E-37 | 163.91 |
|  | rs7583027 | C | A | 2.90E-03 | 4.62E-04 | 3.30E-10 | 39.47 |
|  | rs7596240 | G | A | 2.90E-03 | 4.98E-04 | 6.10E-09 | 33.79 |
|  | rs761357 | T | A | 2.88E-03 | 4.58E-04 | 3.40E-10 | 39.41 |
|  | rs7649344 | C | T | -2.62E-03 | 4.45E-04 | 4.00E-09 | 34.60 |
|  | rs76518703 | G | A | -9.78E-03 | 1.04E-03 | 5.60E-21 | 88.29 |
|  | rs76930710 | C | T | -6.29E-03 | 1.15E-03 | 4.20E-08 | 30.06 |
|  | rs7705526 | A | C | -2.90E-03 | 4.78E-04 | 1.40E-09 | 36.68 |
|  | rs772920 | G | C | 3.59E-03 | 4.69E-04 | 1.90E-14 | 58.60 |
|  | rs7768019 | G | C | -7.93E-03 | 5.14E-04 | 1.20E-53 | 237.76 |
|  | rs7850258 | G | A | 9.90E-03 | 4.70E-04 | 1.90E-98 | 443.46 |
|  | rs7936397 | A | G | -2.94E-03 | 4.98E-04 | 3.60E-09 | 34.84 |
|  | rs79490353 | C | T | 1.03E-02 | 1.41E-03 | 2.30E-13 | 53.77 |
|  | rs8054578 | G | A | -3.41E-03 | 5.30E-04 | 1.20E-10 | 41.39 |
|  | rs8093850 | G | A | 2.79E-03 | 4.78E-04 | 4.90E-09 | 34.24 |
|  | rs897586 | A | G | -3.49E-03 | 4.63E-04 | 5.10E-14 | 56.71 |
|  | rs9277569 | T | C | 8.32E-03 | 7.08E-04 | 7.60E-32 | 137.93 |
|  | rs9291444 | T | C | 3.75E-03 | 4.44E-04 | 3.30E-17 | 71.15 |
|  | rs9347170 | T | C | -5.60E-03 | 4.68E-04 | 4.30E-33 | 143.60 |
|  | rs9497965 | T | C | 3.89E-03 | 4.51E-04 | 5.80E-18 | 74.59 |
|  | rs9511151 | A | G | -6.24E-03 | 4.67E-04 | 1.10E-40 | 178.44 |
|  | rs9697210 | A | G | -3.68E-03 | 6.29E-04 | 5.00E-09 | 34.20 |
|  | rs970987 | A | C | -3.90E-03 | 4.70E-04 | 1.00E-16 | 68.96 |

AA, alopecia areata; AGA, androgenetic alopecia; Hypo, hypothyroidism; SNP, single nucleotide polymorphism; EA, effect allele; OA, other allele; SE, standard error.
